# Photoplethysmography based atrial fibrillation detection: a review

**DOI:** 10.1038/s41746-019-0207-9

**Published:** 2020-01-10

**Authors:** Tania Pereira, Nate Tran, Kais Gadhoumi, Michele M. Pelter, Duc H. Do, Randall J. Lee, Rene Colorado, Karl Meisel, Xiao Hu

**Affiliations:** 1 Department of Physiological Nursing, University of California, San Francisco, CA USA; 2 David Geffen School of Medicine, University of California, Los Angeles, CA USA; 3 Cardiovascular Research Institute, Department of Medicine, Institute for Regeneration Medicine, University of California, San Francisco, CA USA; 4 Department of Neurology, School of Medicine, University of California, San Francisco, CA USA; 5 Department of Neurosurgery, School of Medicine, University of California, Los Angeles, CA USA; 6 Department of Neurological Surgery, University of California, San Francisco, CA USA; 7 Institute of Computational Health Sciences, University of California, San Francisco, CA USA

**Keywords:** Diagnosis, Risk factors

## Abstract

Atrial fibrillation (AF) is a cardiac rhythm disorder associated with increased morbidity and mortality. It is the leading risk factor for cardioembolic stroke and its early detection is crucial in both primary and secondary stroke prevention. Continuous monitoring of cardiac rhythm is today possible thanks to consumer-grade wearable devices, enabling transformative diagnostic and patient management tools. Such monitoring is possible using low-cost easy-to-implement optical sensors that today equip the majority of wearables. These sensors record blood volume variations—a technology known as photoplethysmography (PPG)—from which the heart rate and other physiological parameters can be extracted to inform about user activity, fitness, sleep, and health. Recently, new wearable devices were introduced as being capable of AF detection, evidenced by large prospective trials in some cases. Such devices would allow for early screening of AF and initiation of therapy to prevent stroke. This review is a summary of a body of work on AF detection using PPG. A thorough account of the signal processing, machine learning, and deep learning approaches used in these studies is presented, followed by a discussion of their limitations and challenges towards clinical applications.

## Introduction

Atrial fibrillation (AF) is an abnormal cardiac rhythm characterized by a disorganized atrial activity. AF is recognized in the electrocardiogram (ECG) as an irregularly irregular rhythm lasting more than 30 s, with no discernible P-waves preceding the QRS complex.^1^ AF prevalence is age, gender, and race dependent.^2^ It is particularly high in the elderly population, reaching 10–17% in subjects 80 years and older.^3^ In addition, AF is more prevalent in males and in the white population.^3^ AF is associated with significant morbidity and mortality. One in five strokes is associated with AF and one-third of cardiac arrhythmias hospitalizations are due to AF-related complications. AF has been associated with a twofold increase in the risk of death.^4^ Additionally, the aging population in the US and worldwide is leading to a markedly increasing AF prevalence^3,5^.

The high prevalence of asymptomatic AF has significant clinical implications on the diagnosis and management of AF.^6^ Intermittent ECG evaluation during clinical visits has a low likelihood of detecting paroxysmal AF. Continuous monitoring would increase the chances of AF detection, thereby allowing appropriate primary and secondary stroke prevention strategies to reduce the high morbidity and mortality of stroke.

For patients with acute ischemic stroke or transient ischemic attack, approximately 10% will have new AF detected during their hospital admission.^7–9^ Continuous ECG monitoring for 30 days is recommended in case of an embolic stroke of undetermined cause (cryptogenic).^9^ Novel non-intrusive approaches for cardiac rhythm monitoring can potentially enable early and accurate detection of asymptomatic paroxysmal AF and create a shift in AF management.^10,11^ Especially for asymptomatic AF cases, new tools that allow the AF detection will help make the appropriate clinical decisions.^10^

Photoplethysmography (PPG) has emerged as a low-cost and non-intrusive modality for continuous monitoring of heart rate. A variety of wearable devices offer PPG-based monitoring, including smartphones and smartwatches. A photoplethysmogram is a pulse pressure signal resulting from the propagation of blood pressure pulses along arterial blood vessels. Measured on the periphery, it carries rich information about the cardiac activity, cardiovascular condition, the interaction between parasympathetic and sympathetic nervous systems, and hemoglobin level.^12^ Many physiological parameters can be derived from PPG, including oxygen saturation, heart rate, blood pressure, and cardiac output.^13^ These capacities of PPG open the door to develop new ambulatory diagnosis tools enabling early screening of heart conditions, including arrhythmia.^14^

This review provides an account of the approaches used in PPG-based AF detection. A brief overview of the technology behind PPG is first presented, followed by a summary of methods and algorithms developed for PPG-based AF detection. Recognizing the importance of using PPG to detect AF at scale, the motivation of this review is to guide the future development of algorithms towards clinical-grade applications.

## Photoplethysmography

### PPG signal

PPG waveform is generated during a cardiac cycle and typically measured at a peripheral site. Therefore, it is essentially a pulse pressure waveform that originates from the heart contraction and propagates through the vascular tree. As blood flow is controlled by neural, cardiac, and respiratory interactions, various physiological parameters could theoretically be extracted from analyzing a PPG signal.^15^ For this reason, the PPG signal has rich information about physiological conditions.^13^

PPG waveforms have typical morphological components corresponding to landmark events in the cardiac cycle. During the contraction of the left ventricle, blood is ejected out of the heart and propagates along the arterial tree, this corresponds to the initial positive slope of a PPG pulse. The systolic peak marks the maximum of the waveform. A decrease in amplitude following the systolic peak is marked by a local minimum, or the dicrotic notch, which corresponds to the closing of aortic valves separating the systolic and diastolic phases. In some cases, a third peak following the dicrotic notch can be identified. It corresponds to a reflected component of the forward wave from various reflection sites including vessel bifurcations.^16^

### Clinical parameters

One primary clinical application of PPG is arterial blood oxygen saturation (SpO2) estimation through pulse oximetry.^17^ SpO2 is defined as the percentage of oxygen saturation in the arterial blood, which can be measured by the ratio of oxygenated hemoglobin concentration to the total hemoglobin concentration, with a normal range between 97% and 98%.^18^ Recently, new applications of PPG have emerged for the continuous estimation of valuable cardiovascular parameters in ambulatory settings. Heart rate, blood pressure, and respiratory rate could be closely monitored for fitness or health assessment.^19^ Advanced diagnostic applications of PPG were also envisaged. Cardiac function, arterial stiffness, autonomic nervous system (ANS) responses, and apnea are among conditions that could potentially be detected or evaluated using PPG.

Changes in blood volume are synchronous with the heart beats, such synchrony is manifested by the concordance of inter-beat intervals (RR intervals) measured in PPG and time-synchronized ECG.^20^ Heart rate variability (HRV) is an indirect measurement of ANS, and it has also been considered as a surrogate parameter of the interaction between the brain and cardiovascular system.^21^ HRV metrics can be derived from analyzing RR intervals in time and/or frequency domain as well as using nonlinear dynamic analysis approaches.^22^ Respiratory rate is one of the fundamental vital signs and can be determined from the time–frequency representation of a PPG signal.^23^

Some hemodynamic parameters such as augmentation index (AIx) and pulse wave velocity (PWV) are important biomarkers of arterial stiffness, which is a direct cause of hypertension and a major risk factor for cardiovascular events such as myocardial infarction and stroke. Both AIx and PWV could be derived from PPG,^24,25^ Subendocardial Viability Ratio (SEVR %) and Ejection Time Index (ETI) are two hemodynamic parameters used in the evaluation of cardiac workload that can be estimated with PPG analysis.^25^ Additionally, some studies claim that arterial blood pressure could be estimated using advanced analysis of PPG.^17^

### Modes of PPG measurement

A PPG signal has two main components: a quasi-static direct current (DC) component, which represents light reflected/transmitted from static arterial blood, venous blood, skin and tissues; and pulsatile alternate current (AC) component which arises from modulation in light absorption due to changes in arterial blood volume. PPG measurement can be carried out using two modes: transmission and reflectance. In transmission mode, the light transmitted through the medium is detected by a photodetector (PD), which is positioned in the opposite site of the light source. The sensor must be located on the body at a site where transmitted light can be detected. The measurement site is limited to the extremities of the body, such as the fingertip or earlobe. The greatest disadvantage of the transmission mode is the location of the device that can interfere with daily routine movements.^26^ In reflectance mode, the PD detects light that is back scattered or reflected from tissues, bone, and/or blood vessels, which means the light source and PD are positioned on the same side. Unlike the transmission mode, the measurement sites are not restricted to any particular location, which facilitates a user-friendly monitoring approach. The wrist, forearm, ankle, and forehead are common measurement sites.^27^

Since the basic form of PPG technology requires only a few optoelectronic components (a light source and a PD: to measure the variations on the light reflected/transmitted by the tissues), it can be easily and inexpensively incorporated in various digital devices such as watches, smartphones, or wearables.^28^ The ubiquitous availability of PPG in a wide range of wearable digital devices has motivated the search for new applications and the development of novel biomedical solutions.

## PPG-based AF detection

**Fig. 1:**
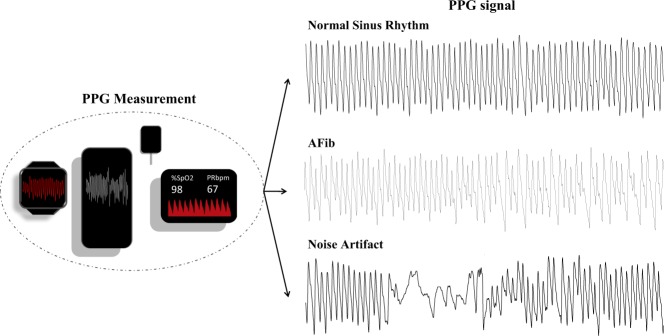
PPG signal acquired using a wearable device and typical waveforms representing NSR, AF, and noise artifact.

In a PPG signal, AF is manifested as varying pulse-to pulse intervals and pulse morphologies. On the other hand, a normal sinus rhythm (NSR) is recognizable through regularly spaced PPG pulses with similar morphologies between consecutive pulses. Recognizing an arrhythmia in a PPG signal can sometimes be challenging in the presence of artifacts. Common sources of artifacts are motion and poor sensor contacts. Artifacts can be misinterpreted as physiological abnormalities. Motion artifacts can be identified using accelerometry data. Most modern wearable devices include accelerometry sensors that measure acceleration forces along different spatial directions. It is a common practice to discard PPG contaminated with an artifact. Figure 1 depicts samples of PPG with NSR, AF, and artifact.

ECG remains the gold standard for the electrophysiological definition and recognition of arrhythmias,^1^ including AF diagnosis.^29^ In a recent study, new deep learning approaches achieved cardiologist-level AF detection of 12 types of arrhythmia (F1 score = 0.84 vs F1 score = 0.78) when 91,232 single-lead ECGs from 53,549 patients were analyzed.^30^ Compared to ECG, PPG-based AF detection is more challenging but also rewarding in situations where longer monitoring time and lower cost beyond what ECG offers is needed, e.g., screening AF at scale.

Recent advances in sensor technologies and wearable devices have increased the role that a PPG-based solution could play in the assessment of health status. Electronics capable of recording PPG signals with relatively high signal-to-noise ratio (SNR) may warrant reliable PPG monitoring and screening of arrhythmia.^11,31^

**Fig. 2:**
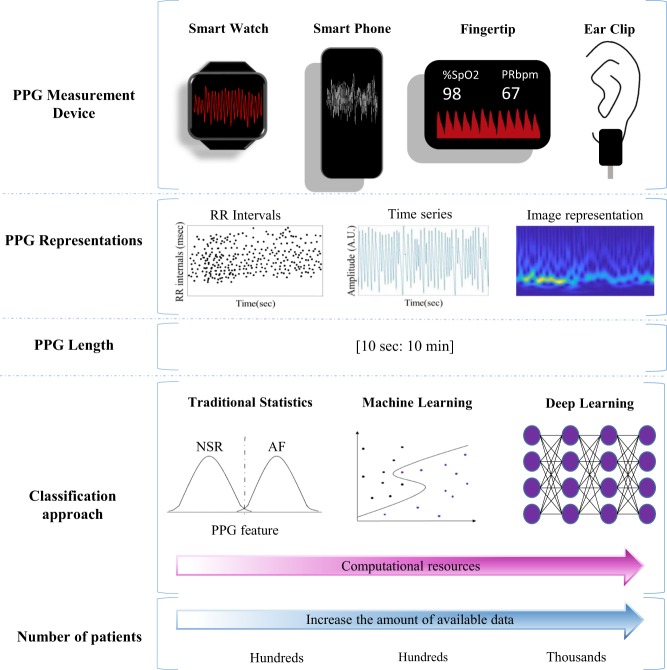
Overview of the main features extracted from PPG signals used in the studies reviewed (see Tables 1–3). SpO2 oxygen saturation, PRbpm pulse rate (beats per minute).

In a typical AF detection algorithm, features (temporal, spectral, or morphological) are extracted from the acquired PPG signal and analyzed by the detection algorithm to inform if an AF rhythm is detected. In some approaches, image representation of the temporal waveform has been considered. The derived image would then be analyzed using conventional image processing or artificial intelligence-based methods (Fig. 2).^32–34^ Traditionally, prominent features were derived from the tachogram (RR intervals) since it is a reliable measure of heart beats.^35^ Realizing that PPG waveforms may carry physiological information beyond heart rate, new features beyond RR intervals were derived.^36^ The use of PPG time series and their images representation (e.g. raw plot of the signal, fast Fourier transform spectrum, or wavelet spectrogram—represented in the Fig. 2 in PPG representation part) were used with promising results in the detection of physiological events,^32,37,38^ Images for PPG representation in Fig. 2 is a general depiction of the format types of information used by the different algorithms.

In the following sections, we review studies of PPG-based AF detection. A body of white papers and peer-reviewed works indexed by PubMed, Scopus, IEEE Xplore, and Web of Science up to June 2019 was selected based on the following search expression: (PPG “*OR*” Photoplethysmography) “*AND*” (atrial fibrillation “*OR*” AF “*OR*” AFib) “*AND*” (detection “*OR*” recognition). Each study is reviewed with respect to the size, the number of patients, and recording settings of data analyzed, the PPG device and site of recording, the AF detection algorithm, and its performance. Figure 2 summarizes the main features examined in these studies, described with more details in Tables 1–3.

**Table 1:** Studies on photoplethysmography-based AF detection using statistical analysis approaches.

Author (year) [ref.]	Number of patients	Dataset features	Age of population	Length PPG segments	Measurement device	Acquisition conditions	Input data	Methodology	Performance results for rhythms detection
Lee et al. (2013)^91^	74	74 prior and after cardioversion + Public databases **(**MIT-BIH AF + MIT-BIH NSR + MIT-BIH Arrhythmia Database)	–	2 min	Video camera of smartphone	Inpatient	RR times series features Time varying coherence functions and Shannon entropy	Derived threshold values of features for best ROC	Acc = 0.9645, Sen = 0.9716, Sp = 0.9539
Nemati et al. (2016)^52^	46	15 with AF 31 non-symptomatic	–	3.5 to 8.5 min	Wrist-worn device Samsung Simband	Inpatient	RR times series features sample entropy with the embedding dimensions *m* = 1, and 2 (SampEn1 and SampEn2), standard deviation, robust standard deviation	Elastic Net logistic model	Acc = 0.95, Sen = 0.97, Sp = 0.94, AUC = 0.99
Bonomi et al. (2016)^50^	16	4 with AF, 1 atrial flutter, 11 NSR	65.2 ± 14.0	30 s	Wrist-wearable sensor— Philips Cardio and Motion Monitoring Module	Outpatient—continuous measurement	RR times series features	First-order 11-state Markov model	Sen = 0.97 ± 0.02, Sp = 0.99 ± 0.03
J. Eckstein et al. (2016)^92^	80	40 with AF 40 Non-AF	80 ± 8 75 ± 7	5 min	Video camera of smartphone	In- and outpatient checkpoint	RR times series features RMSSD and SD1/SD2 index extracted from the Poincare plot	Derived threshold values of features for best ROC	AUC = 0.931, Sen = 0.950, Sp = 0.950
D. McManus et al. (2016)^93^	121	98 with AF 15 with PAC 15 with PVC	66	2 min	Video camera of smartphone	Inpatient	RR times series features RMSSD, Shannon Entropy, Poincare plot	Derived threshold values of features for best ROC	AF**:** Acc = 0.951, Sen = 0.970, Sp = 0.935. PAC: Acc = 0.955, Sen = 0.667, Sp = 0.980. PVC: Acc = 0.960, Sen = 0.733, Sp = 0.976
Shashikumar et al. (2017)^51^	98	45 with AF, 53 with other rhythms (ARR)	–	30 s	Wrist-worn device Samsung Simband	Inpatient	PPG image spectral representation of wavelet transform—features obtained from the CNN + RR times series features (sample entropy, standard deviation, robust version of the standard deviation, min and the max of the sample entropy features)	Elastic net logistic model	Acc = 0.918, AUC = 0.95
T. Conroy et al. (2017)^94^	77 Test: 34	44 healthy subjects, 33 with AF, 13 healthy subjects, 21 with AF	38 ± 12, 64 ± 11, 45 ± 17, 68 ± 11	5 min	Single earlobe PPG sensor	Inpatient	RR times series features. Coefficient of variation, standard deviation, average of the difference in beat-to-beat: pNN35	Derived threshold values of features for best ROC	Acc = 0.952, Sen = 0.909, Sp = 0.909
Tang et al. (2017)^48^	666 stroke patients	150 with AF, 516 Non-AF	74.5 ± 12.8, 66.3 ± 14.8	1 min, 2 min, 10 min	Bedside monitor	Inpatient	RR times series features. Time domain: mean, standard deviation, and RMSSD. Frequency domain: low-frequency range (LF), power in the high-frequency range (HF), and the ratio of LF and HF. Nonlinear analytical methods: Shannon entropy, and turning point ratio	Logistic regression analysis	1-min: AUC = 0.949, 2-min: AUC = 0.972, 10-min: AUC = 0.973
Bashar et al. (2018)^64^	200	–	Older than 45 years old	30 s	Video camera of smartphone	Outpatient—checkpoint	Noise/movement detection: Variable frequency complex demodulation. AF detection: RR times series features (RMSSD, Shannon entropy and sample entropy)	Noise/movement detection: thresholds. AF detection: Support vector machines	Acc = 0.9116
Chong et al. (2018)^65^	99	88 patients with AF prior and after cardioversion 11 health subjects	–	2 min	Video camera of smartphone	Outpatient—checkpoint	Noise/movement detection: Signal slope changes, turning point ratio changes, and kurtosis change. AF detection: RMSSD and Shannon Entropy (ShE)	Noise/movement detection: thresholds. AF detection: thresholds	Acc = 0.9667, Sen = 0.9765, Sp = 0.9714
Tarniceriu et al. (2018)^49^	29	15 NSR, 14 with AF	67.5 ± 10.7, 74.8 ± 8.3	20 consecutive RR	Wrist-worn device PulseOn Ltd.	Inpatient	RR times series features	Markov model	Sen = 0.9845, Sp = 0.9913
H.M. de Morree et al. (2018)^74^	27	8 AF 19 non-AF	69 ± 101, 67 ± 13	120 s	Wrist-worn device Philips Cardio	Outpatient—continuous measurement	RR times series features Shannon entropy, RMSSD, normalized RMSSD, pNN40, pNN70, sample entropy, and coefficient of sample entropy	Derived threshold values of features for best ROC	Acc = 0.981, Sen = 0.984, Sp = 0.980

RR R to R interval, *NSR* normal sinus rhythm, *ARR* other arrhythmias, *VA* ventricular arrhythmias, *AUC* area under the curve, *Acc* accuracy, *Sen* sensitivity, *Sp* specificity, *PVC* premature ventricular contractions, *PAC* premature atrial contraction, pNN35/pNN40/pNN70 percentage of differences of successive RR that exceeded 35 or 40 or 70 ms by the total number of RR intervals

**Table 2:** Studies on photoplethysmography based AF detection using ML approaches.

Author (year) [ref.]	Number of patients	Dataset features	Age of population	Length PPG segments	Measurement device	Acquisition conditions	Input data	Methodology	Performance results for rhythms detection
Shan et al. (2016)^56^	468 stroke patients	–	–	2 min	Bedside monitor	Inpatient	RR times series features. Mean, median, standard deviation, RMSSD, power in very low-frequency range, low frequency (LF), high frequency (HF), ratio of power in LF and HF (LF/HF), multi-scale entropy, Shannon entropy, Turning point ratio	Support vector machines	AUC = 0.971, Sen = 0.942, Acc = 0.957
M. Lemay et al. (2016)^57^	20		–	10 s	PPG wrist- based device	Inpatient	RR times series features mean, minimum, median, and interquartile range of RR	Support vector machine	Acc = 0.9385
V. Corino et al. (2017)^58^	70	30 with AF, 9 ARR, 31 NSR	40 ± 17, 76 ± 9, 65 ± 15	2 min	Empatica E4 wristband	Inpatient	RR times series features 24 features: spectral analysis, variability and irregularity analysis, PPG-waveform features	KNN classifier	NSR: Sen = 0.773, Sp = 0.928. AF: Sen = 0.754, Sp = 0.963. ARR: Sen = 0.758, Sp = 0.768
T. Schack et al. (2017)^95^	326	20 with AF, 294 of NSR, 12 of Noise	–	20 s	Video camera of smartphone	Inpatient	RR times series features Mean, median, standard deviation and the mean absolute deviation (MAD); RMSSD; normalized RMSSD; Shannon entropy. PPG-waveform features: mean, median, SD and MAD, crest time, peak rise height, fall height, waveform width, cross-correlation of consecutive pulse segments, very low frequency, low frequency, high frequency and quotients of these spectral powers	Support vector machines	Perfect detection of AF
S. Fallet et al. (2019)^60^	17	AF NSR ventricular arrhythmias (VA)	57 ± 13	10 s	Wrist-worn device	Inpatient	RR times series features: mean, standard deviation, median, interquartile, minimum, maximum, RMSSD. PPG-waveform features: adaptive organization index, variance of the slope of the phase difference, permutation entropy, spectral entropy, fractional spectral radius, and spectral purity index).	Decision trees	AF vs NSR: Acc = 0.981, Sen = 0.997, Sp = 0.924. AF vs VA: Acc = 0.959, Sen = 0.981, Sp = 0.887. AF vs (NSR&VA): Acc = 0.950, Sen = 0.962, Sp = 0.928

*RR* R to R interval, *NSR* normal sinus rhythm, *ARR* other arrhythmias, *VA* ventricular arrhythmias, *AUC* area under the curve, *Acc* accuracy, *Sen* sensitivity, *Sp* specificity, *PVC* premature ventricular contractions, *PAC* premature atrial contraction

**Table 3:** Studies on photoplethysmography based AF detection using DL approaches.

Author (year) [Ref]	Number of patients	Dataset features	Age of population	Length PPG segments	Measurement device	Input data	Acquisition conditions	Methodology	Performance results for rhythms detection
Aliamiri and Shen(2018)^75^	19		—	30 s	Samsung gear device	PPG segment	—	Quality classification: CNN AF. detection: Convolution-Recurrent Hybrid Model (CRNN)	Acc = 0.9819, AUC = 0.9967
Tison et al. (2018)^72^	Train: 9750. Test set 1: 51. Test set 2: 1617	Train: 347 with AF, 8216 no AF	Train: 42 ± 12. Test set 1: 66.1 ± 10.7	5 s	Wrist-worn device Apple watch	RR times series features	Test set 1: Inpatient Test. set 2: Outpatient checkpoint	Neural network of 8-layers	Test set 1: AUC = 0.97, Sen = 0.980, Sp = 0.902. Test set 1: AUC = 0.72, Sen = 0.677, Sp = 0.676
M. Poh et al. (2018)^73^	Train: 3373. Test: 1013	Train: Public databases (MIMIC-III critical care database + Vortal dataset from healthy volunteers + IEEE-TBME PPG Respiratory Rate Benchmark dataset)	Test group: 68.4 ± 12.2	17 s	Smartphone	PPG segment	Outpatient	CNN architecture with six dense blocks	Overall Acc = 0.961. Noise: Sen = 0.970, Sp = 1. NSR: Sen = 0.991, Sp = 0.982. ARR: Sen = 0.722, Sp = 0.988. AF: Sen = 0.976, Sp = 0.965
I. Gotlibovych et al. (2018)^97^	Train: 42. Test: 11	Train: 29 with AF 13 NSR. Test: 7 with AF 4 NSR	37–85 years old	—	Wrist-worn prototype fitness tracker device	PPG segment	Inpatient + Outpatient NSR: asleep continuous measurement	Convolutional-recurrent neural network	AUC = 0.999, Sen = 0.999, Sp = 0.998
M. Voisin et al. (2019)^81^	81	Train + validation**:** 19 AF/32 NSR. Test: 10 AF/20 NSR	—	30 s	Wrist-worn device Samsung Simband	PPG segment	Outpatient—continuous measurement	1D ResNet	AUC = 0.949
S. Shashikumar et al. (2018)^32^	Train: 2850. Test: 97	Test: 44 AF and 53 ARR	Train: 47 ± 25 years. Test: 18–89 years old	30 s	Wrist-worn device Samsung Simband	ECG image-based– training. PPG image-based - test	Outpatient—checkpoint	Bidirectional Recurrent Neural Network (BRNN) with transfer learning	AUC = 0.97, AUCpr = 0.97, Sp = 1.0, Acc = 0.95
S. Kwon et al. (2019)^96^	75		63 ± 7.8	30 s	PPG fingertip	PPG segment	Inpatient	1-Dimensional convolutional neural network. Recurrent neural network	Acc = 0.9758, AUC = 0.998. Acc = 0.9715, AUC = 0.996

*RR* R to R interval, *NSR* normal sinus rhythm, *ARR* other arrhythmias, *VA* ventricular arrhythmias, *AUC* area under the curve, *Acc* accuracy, *Sen* sensitivity, *Sp* specificity, *PVC* premature ventricular contractions, *PAC* premature atrial contraction, *AUCpr* area under the precision–recall curve

### Performance metrics

AF detection algorithms can be evaluated using several performance metrics. It is common for many studies to report sensitivity, specificity, and accuracy. Sensitivity is defined as the probability to detect true AF events, while the specificity measures the proportion of actual Non-AF instances correctly identified as such. Accuracy is a balanced metric of sensitivity and specificity. The accuracy of an AF detection algorithm is its ability to differentiate between AF and Non-AF cases.^39^ Generally, accuracy is the most common reported metric, along with the area under the curve (AUC) of the receiver operating characteristic (ROC). A ROC for differentiating AF *vs* Non-AF is generated by plotting sensitivity *vs* (1-specificity) at different classification thresholds. AUC is a measure of how well AF cases ranked higher than Non-AF cases. Since AF has a low prevalence it is generally required that PPG-based AF detectors show high precision (positive predictive value). Rather than reporting the AUC, the area under the precision-recall curve (AUPRC) is an alternative metric suitable for highly imbalanced data (i.e. low prevalence).^40^ In general, any reported performance metric should take into account the low prevalence of AF and be evaluated on an independent test dataset.

### AF detection studies

Studies were split into three groups based on the approaches undertaken to build an AF detector: traditional statistical analysis, machine learning (ML), and deep learning (DL) methods. In traditional statistical analysis, statistical metrics are derived from PPG signals, and classification thresholds were estimated to distinguish between AF and Non-AF segments. ML techniques call for the extraction of pre-selected features, a process that can be quite manual, labor-intensive, and can usually benefit from incorporating complex physiological knowledge. An ML classifier is then built upon extracted features from training data samples. DL approaches require less manual feature engineering than conventional ML since DL incorporates automatic features representation process of input data. Recently, there was a significant focus on DL methods driven notably by technological advancement in computational power and the acclaimed success in computer vision applications.^41,42^

### Statistical analysis approaches

Statistical models for AF detection are built using the thresholds for a set of features extracted from the RR-interval time series of well-annotated and publicly available ECG databases, such as MIT-BIH atrial fibrillation, MIT-BIH normal sinus rhythm, or MIT-BIH arrhythmia database.^43–45^ Specifically, features were first extracted from the RR-interval time series of pre-annotated ECG waveforms. The histograms of each feature were analyzed respectively with or without the presence of AF and other cardiac rhythms in order to define the threshold that best separates the rhythm classes. These thresholds were then applied to the same RR time series-based features extracted from PPG signals.^46,47^ Other arrhythmias (i.e., premature ventricular contractions, and premature atrial contraction) could also be detected similarly in a sequence of binary classifications.^36^

Other statistical approaches can also be applied to classify between AF and Non-AF such as logistic regression.^48^ Logistic regression models use the logistic function, instead of a straight line or a hyperplane, to fit output the probability between 0 and 1 (corresponding to Non-AF and AF). Markov model is another statistical tool that could be used for AF detection. RR-interval time series features are used in this model to define the distributions that best fit the data, and the probability for various rhythms can be drawn from these distributions,^44,49,50^ Elastic net is a regularization method for regression and classification models. Elastic net performs both variable selection and regularization in order to enhance the prediction accuracy and interpretability of the logistic regression model. Regularization approaches were successfully applied to improve the performance of AF detection.^51,52^

Table 1 summarizes a selection of PPG-based AF detection studies which used statistical models. Different study aspects are shown to depict the patient population and datasets used, the features and methods, the context (inpatient vs outpatient), and the performance results.

#### Machine learning approaches

ML has been used for AF detection with interesting results. ML techniques require extensive domain expertise to design features suitable for a comprehensive representation of PPG waveforms and the detection of class-differentiating patterns. Features commonly extracted from PPG time series are morphological descriptors, time domain statistics, frequency domain statistics, nonlinear measures, wavelet based measures, and cross-correlation measures.^53–60^

There were generally three main ML approaches used in the reviewed studies: k-nearest neighbors (KNN), support vector machine (SVM), and decision trees (DT). KNN classification is a relatively simple clustering technique where a sample is classified by a plurality vote of its neighbors and assigned to the class based on the most common class among its *k* closest neighbors.^61^

SVM finds a hyperplane that separates two classes with a high margin that maximizes the distances between nearest data points from each class. SVMs prove to be successful in nonlinear classification problems by mapping non-separable features into a higher dimensional space, a procedure known as the kernel trick which uses kernel functions such as Radial Basis Function (RBF) or polynomial.^62^

In DT approaches, the training set is continuously split according to a chosen feature. A feature tree can be explained by two entities, namely decision nodes and leaves. The leaves are the decisions or final outcomes. And the decision nodes are where the data is split.^63^ The objective is to find in each decision node of the tree, the best attribute allowing to diminish as much as possible the overlapping of classes. The classification starts from the root, and it evaluates the relative attribute and it takes the branch corresponding to the outcome. This process is repeated until a leaf is encountered and a sample is assigned the class labeling the leaf.

Some studies used a combination of threshold-based and ML approaches. For example, the thresholds of some features were first used to exclude poor pulses, then an ML model was built for the detection of AF in the clean pulses.^64,65^ Table 2 is a chronological summary of the selected ML studies and the reported performance results. All the studies reported in the Table 2 were based in short length of PPG segment, with maxima of 2 min.

#### Deep learning approaches

DL has recently emerged as a powerful method for the detection of abnormalities in physiological signals, encouraging applications of arrhythmia detection from ECG and PPG, including AF detection. Unlike ML, deep learning models automatically learn feature representations, sparing the tedious task of feature crafting. DL uses a neural network, a set of interconnected layers of computational nodes. The most common DL approaches used for AF detection are based on Convolutional Neural Networks (CNN). CNN was applied in automatic feature extraction and in classification problems. Some studies used CNNs only for automatic feature extraction.^51^ In one study, an aggregated model of two serially connected CNNs was proposed, where the former detects clean segments from which the latter identifies instances of AF.^51^ Some DL models were trained with hybrid input data (i.e., time series and images) in order to capture a wide range of features spanning more than one domain.

Training a DL model from scratch requires a large amount of labeled training data and generally poses a major constraint in biomedical applications due to the limited amount of labeled data. A possible solution to overcome this limitation is transfer learning where the task is to fine-tune a sophisticated pre-trained DL.^66^ The required number of layers and the complexity of fine-tuning depend on specific applications.^67^ In ref. ^32^ a pre-trained ECG-based CNN model was used to detect AF from PPG segments by fine-tuning the network using a small set of labeled PPG segments.

Table 3 is a chronological summary of the selected DL studies and the reported performance results. Notably, all studies were based on relatively short segments (less than 10 min), mainly due to the low yield of AF events in PPG data. Table 3 is a chronological summary of the selected DL studies and the reported performance results. Notably, all studies were based on relatively short segments (<10 min), mainly due to the low yield of AF events in PPG data. Many of recent studies were developed and tested using ambulatory data (outpatients). Such data setting is the closest to real conditions where developed solutions would be applied.^68^ Training and testing algorithms on real-world data is crucial to assess their true performance and evaluate their readiness for commercial use. For this reason, most of the studies in Table 3 are based on outpatient data.

Recently, large-scale AF screening studies have been performed by the leading tech companies, in order to test the performance of the most recent PPG-based smart devices.

In Huawei Heart study a continuous PPG was monitored in a cohort of 187,912 patients using a smart-device in ambulatory conditions. Participants were monitoring for at least 14 days with a wristband or wristwatch and a 60-s PPG signal was continuously measured at every 10 min.^69^ Only individuals identified with “suspected AF” were assessed by using ECG. Results showed 87.0% of cases were AF with positive predictive value (PPV) of 91.6%.^69^ The algorithm for AF detection was not described; however, morphology and frequency analysis of the pulse waveform was used to identify the AF events according to a previous publication.^70^

Apple Heart Study enrolled 419,093 participants in a prospective study.^71^ PPG was monitored by Apple Watch, a subsequent ambulatory ECG patch was used for cases initially identified as AF using a proprietary PPG-based algorithm. Therefore, this study was not included in this review.

Both studies claim they demonstrate the ability of a smart devices to screen AF events. Due to the study design of both studies, they cannot assess the sensitivity of PPG-based AF detection, since only participants identified with AF events (irregular pulse notification) received an ECG monitoring system.

## Main current challenges

While the performance results summarized in Tables 1–3 suggest that PPG can be an alternative to ECG for AF detection, it remains that in real-world applications, PPG-based AF detection could be limited by a number of factors.

### Other cardiac arrhythmias

The presence of different cardiac rhythms within a recording poses a challenge for AF detection. For some of the works reviewed, control data only contain NSR.^46,47,72^ Statistical methods for AF detection are usually limited to distinguishing between AF and NSR since other arrhythmias will likely present a distribution with mixed characteristics from both distributions of AF and NSR cases. ML and DL methods are generally more robust than statistical methods in distinguishing AF from a variety of rhythms. With DL expected to be on the rise for the next years, AF detection can benefit from new and more reliable DL algorithms. Such advancement will likely require very large training datasets of comprehensive cardiac rhythmic morphologies. New algorithms are expected to be better in discriminating AF and AF-mimic rhythms leading to high and perhaps clinical-grade performance levels.^30^ New algorithms are also expected to demonstrate robustness to noise, inter- and intra-subject variabilities, and to other variables currently posing issues to existing methods.

### Corrupted signals

A significant limitation with PPG signals is motion artifacts. Artifacts in PPG signals can generate fluctuations and distortions that complicate the detection of cardiac components. Sources of motion artifact usually include movement of the PPG sensors either on the skin or loose contact with the skin, and deformation of the illuminated tissue volume by dynamic variations in sensor contact-pressure. Specifically, for AF detection, these problems present a major challenge. PPG signals with AF and motion artifacts can both have similar characteristics of irregular pulse-to-pulse intervals. This situation can lead sinus rhythm signals corrupted by motion artifacts to be incorrectly detected as AF and vice versa.^65,73^ The most common approach to deal with this issue is to simply discard the corrupted segments and use only the clean parts of PPG signals.^49,50,58,60,64,74^ Some of the works followed a two-step approach: first, to identify motion artifacts by using accelerometer data, or by performing PPG signal quality assessment; and second, to perform AF detections with only good quality signals.^51,75^ This often implies loss, and in some cases, a huge part of the signals acquired. One study shows that almost 40% of collected PPG signals were reported unreliable.^50^ To overcome this limitation, one can improve the wearables in order to be more robust in detecting motion artifacts and to develop powerful methods to recover poor signals. Recent progress in flexible and stretchable sensors could help to enhance the SNR. Novel flexible sensors for transmission and reflection-mode pulse oximetry show a higher SNR due to a reduction in ambient noise.^76^

Algorithms such as the independent component analysis (ICA),^77^ Kalman filtering, wavelet denoising,^78^ and empirical mode decomposition^79,80^ were proposed for removing artifacts in PPG signal; however, these techniques were mainly proposed for scenarios with weak noise.

Ideally, AF detection models can continuously and accurately detect AF episodes in PPG collected in an ambulatory setting, without discarding PPG segments and being robustness to motion artifacts.^81^ Other DL techniques can be interesting options to solve artifact issues. For example, Generative Adversarial Networks (GAN) is a technique that combines two different neural networks together into a single pipeline—generative and discriminative neural networks. GANs have been used to recover information from images^82^ and biomedical signals,^83^ and can be an interesting option to recover the PPG signal.

### Data annotation

The methods used for AF classification presented in Tables 1–3 are based on supervised methods where data were labeled, using a ground truth (often based on simultaneously recorded ECG). Manual annotation is usually very time consuming, expensive, and laborious. Other annotation methods, such as the use of other devices to identify ground truth, are usually not reliable since the patient needs to use more devices, in a continuous and synchronized way and there are no “perfect” devices with perfect sensitivity and specificity in detecting AF. Active learning is a strategy that aims to ease the data labeling process by automatically deciding which instances should be labeled. Instead of annotating an entire dataset, active learning minimizes manual labeling effort, creating annotated datasets in an efficient way,^84,85^

Another disadvantage of the manual annotation is inherent inter-rater variability reflected in inter-rater disagreements coefficients (such as Kappa scores). Ideally, heuristic rules defining the annotation process are precise enough to warrant an ideal inter-rater agreement. However, in AF detection as in most physiological patterns, data complexity and pattern variability among and within patients render high inter-rater agreement difficult to reach. Such disagreement leads to a labeling noise that translates into a bias that the AF detection algorithm needs to deal with. Mislabeled training samples can potentially affect the performance of supervised classifiers.^86^ Generally, relatively small proportions of noisy labels are tolerated in large training datasets but high proportions of noisy labels can severely degrade the performance of a classifier.^87,88^ At the data level, mislabeling can be mitigated using voting from multiple annotators. At the algorithm level, some DL models were shown to be resistant to relatively small levels of label noise.^87^

### Other challenges

DL algorithms have started to be applied in PPG-based AF detection and in general, showed superior performance. However, the computational resources required for low-latency real-time inference can be a huge engineering challenge to actually deploy the DL algorithm on wearable devices. Wearables have limited energy and constrains for battery size and heat dissipation. Due to these limitations, some of PPG devices were combined with computation-intensive applications on smartphones.^89^

In addition, the interpretability of black-box DL algorithms can be a challenge for doctors to perform diagnosis based on results from those algorithms. Many machine learning algorithms used in the clinical field are essentially black boxes, which make predictions without giving any clinical explanation. The clinical community points out that the accuracy achieved by the ML algorithm is not enough for accepting ML-enabled technology.^90^ Explainable ML algorithms are emerging with interpretable models, which can give information about which aspect of input data contributes more to the final prediction. The most recent approaches for AF detection showed that the model seems to focus on systolic and diastolic peaks and slopes.^81^

A general limitation in the medical field and also in the studies of AF detection is the fact that models take into consideration a small group of patients from one medical center. The models should be developed using data from multiple medical centers in order to ensure that all the population heterogeneities were represented in the sample used for the study.

## Conclusions

A review of statistical and machine learning approaches applied to AF detection using PPG is presented. Although PPG has proven to be a good alternative to ECG for ambulatory real-time and continuous detection of AF, there are challenges remaining to be solved that are currently limiting the expansion of PPG-based AF detection beyond consumer wearables and its application in other clinical applications. Recent advances in computational power and the advent of powerful deep earning algorithms capable of solving complex pattern recognition problems have led to new AF detection methods proving a significant improvement in accuracy, robustness, and reliability compared to earlier approaches. Whether deep learning will ultimately lead to clinical-grade performance levels of PPG-based AF detection remains a question. Wearable devices powered by sophisticated algorithms offering precise and continuous AF detection will provide an excellent opportunity to screen AF at scale as demonstrated in the recent Apple and Huawei studies.
